# Non-structural protein NS3/NS3a is required for propagation of bluetongue virus in *Culicoides sonorensis*

**DOI:** 10.1186/s13071-015-1063-3

**Published:** 2015-09-17

**Authors:** Femke Feenstra, Barbara S. Drolet, Jan Boonstra, Piet A. van Rijn

**Affiliations:** Department of Virology, Central Veterinary Institute of Wageningen UR, Lelystad, The Netherlands; Department of Infectious Diseases and Immunology, Faculty of Veterinary Medicine, Utrecht University, Utrecht, The Netherlands; Arthropod-Borne Animal Diseases Research Unit, Agricultural Research Service, U. S. Department of Agriculture, Manhattan, KS USA; Department of Biochemistry, Centre for Human Metabonomics, North-West University, Potchefstroom, South Africa

**Keywords:** Bluetongue virus, Culicoides, Arbovirus, DISA vaccine, NS3/NS3a, Midge

## Abstract

**Background:**

Bluetongue virus (BTV) causes non-contagious haemorrhagic disease in ruminants and is transmitted by *Culicoides* spp. biting midges. BTV encodes four non-structural proteins of which NS3/NS3a is functional in virus release. NS3/NS3a is not essential for *in vitro* virus replication. However, deletion of NS3/NS3a leads to delayed virus release from mammalian cells and largely reduces virus release from insect cells. NS3/NS3a knockout BTV in sheep causes no viremia, but induces sterile immunity and is therefore proposed to be a Disabled Infectious Single Animal (DISA) vaccine candidate. In the absence of viremia, uptake of this vaccine strain by blood-feeding midges would be highly unlikely. Nevertheless, unintended replication of vaccine strains within vectors, and subsequent recombination or re-assortment resulting in virulent phenotypes and transmission is a safety concern of modified-live vaccines.

**Methods:**

The role of NS3/NS3a in replication and dissemination of BTV1, expressing VP2 of serotype 2 within colonized *Culicoides sonorensis* midges was investigated. Virus strains were generated using reverse genetics and their growth was examined *in vitro*. A laboratory colony of *C. sonorensis,* a known competent BTV vector, was fed or injected with BTV with or without expressing NS3/NS3a and replication in the midge was examined using RT PCR. Crossing of the midgut infection barrier was examined by separate testing of midge heads and bodies.

**Results:**

Although the parental NS3/NS3a expressing strain was not able to replicate and disseminate within *C. sonorensis* after oral feeding, this virus was able to replicate efficiently when the midgut infection barrier was bypassed by intrathoracic injection, whereas the NS3/NS3a knockout mutant was unable to replicate. This demonstrates that NS3/NS3a is required for viral replication within *Culicoides*.

**Conclusion:**

The lack of viremia and the inability to replicate within the vector, clearly demonstrate the inability of NS3/NS3a knockout DISA vaccine strains to be transmitted by midges.

## Background

Arthropod-borne viruses (arboviruses) have a significant social and economic impact on both human and animal health. A majority of all emerging and re-emerging infectious diseases are vector-borne or zoonotic [1, 2]. Combating arboviral diseases requires an interdisciplinary approach, possibly including vector control, surveillance programs and outbreak containment, with prevention of disease by vaccination likely the most promising [3]. Bluetongue virus (BTV, family *Reoviridae*, genus *Orbivirus*) [4] and Schmallenberg virus (family *Bunyaviridae*) [5] are examples of emerging arboviruses in countries with a moderate climate, which are transmitted by *Culicoides* (Diptera: *Ceratopogonidae*) biting midges [6–8]. BTV infection results in a haemorrhagic disease of domestic and wild ruminants called Bluetongue (BT). In cattle, BT is typically subclinical, but infection in sheep can result in severe disease with high mortality [9]. Due to the wide host range, prolonged viremia with often less-severe disease symptoms, and virus spread by midges, outbreak control is difficult using control measures other than vaccination [3].

BTV is a non-enveloped virus with a complex triple-layered capsid containing the ten-segmented double stranded (ds) RNA genome and the replication complex. In addition to seven structural proteins (VP1-7), BTV encodes four non-structural (NS) proteins [10–12]. The virus release mechanism differs for mammalian and insect cells, with insect cells showing non-lytic release, whereas cell lysis is the prominent release mechanism in mammalian cells [13]. Virus particles can leave the cell by budding, acquiring a temporary envelope, but also via disruption of the cell membrane [13, 14].

NS3 and N-terminal truncated NS3a are viroporin-like membrane proteins [15, 16], functional in virus release [17, 18]. NS3/NS3a is expressed in larger amounts in insect cells and is hypothesized to be mainly important for the non-lytic release from these cells [19–23]. NS3/NS3a comprises a long N-terminal domain, two transmembrane domains with a short extracellular domain in between, and a shorter C-terminal cytoplasmic domain [24]. Interaction with the p11 cellular calpactin complex subunit and recruitment of the ESCRT-I TsgI protein [25–27], highlights involvement in membrane trafficking/modification and virus release. Recently, we showed that expression of NS3/NS3a is not essential for BTV replication *in vitro.* However, release of NS3/NS3a knockout mutants from mammalian cells is significantly delayed and release from insect cells is strongly reduced [28].

By inducing an out of frame deletion in BTV Seg-10, encoding NS3/NS3a, using reverse genetics, knockout BTV viruses were generated. A BTV vaccine strain with such a Seg-10 deletion has been described to be a very promising vaccine candidate, named the disabled infectious single animal (DISA) BT vaccine. Sheep have been vaccinated with this virus, and no clinical signs after vaccination were induced. Vaccine virus replicated only locally and sterile protection to virulent BTV infection was induced [29–31]. Since vaccination did not result in detectable viremia of BT DISA vaccine virus, oral uptake of the vaccine by insect feeding is highly unlikely.

**Fig. 1:**
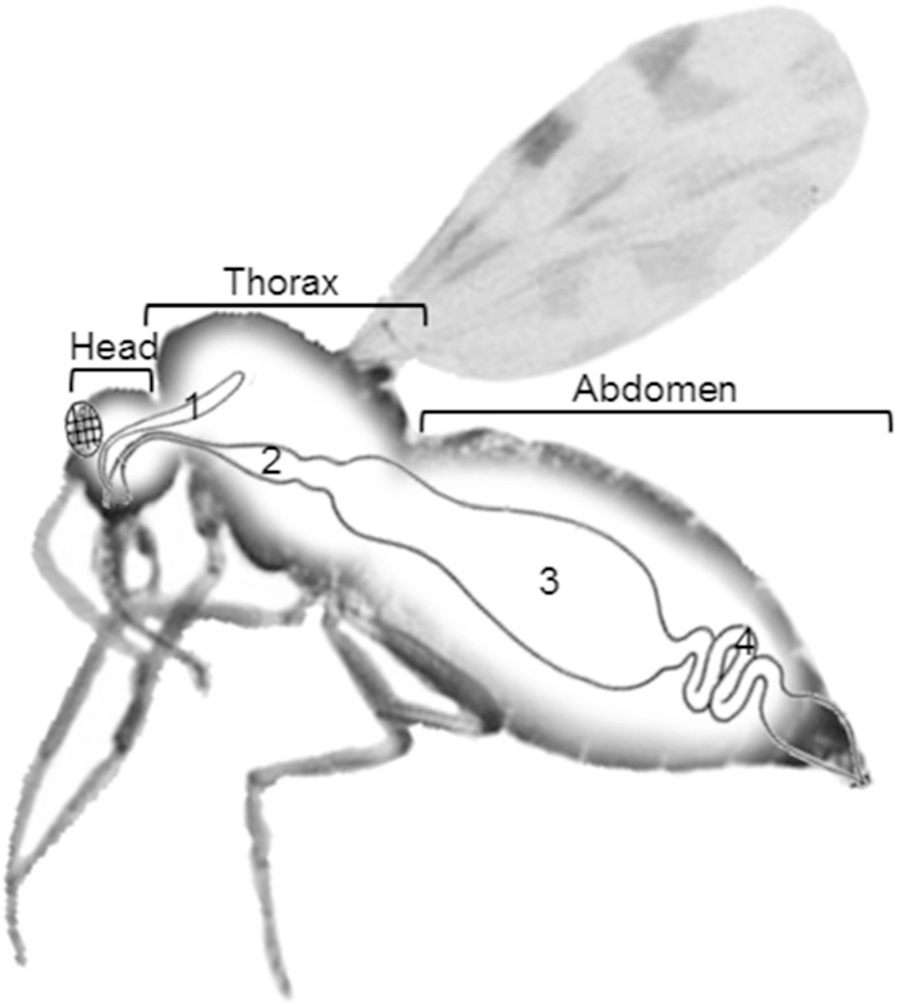
*Culicoides* midge with the several different body parts; head, thorax and abdomen, indicated. Salivary glands (1) are located in the head/thorax. The digestive tract contains the forgut (2) and midgut (3), where food is digested and the hindgut (4), where faeces forms. Important dissemination barriers are located at the level of the midgut and salivary glands. Figure partly adapted from Alan R. Walker (Veterinary Research Laboratory, Kenya)

Oral infection of midges with BTV and subsequent virus transmission to the ruminant host is complex and differs even between individuals of one *Culicoides* species. This leads to variable proportions of individuals within a midge population being susceptible to oral virus infection or capable of virus transmission. Vector arthropods present several ‘barriers’ which could prevent infection, dissemination, or transmission of the arbovirus to the susceptible host. For the *Culicoides* vector, a midgut infection barrier (MIB), a midgut escape barrier (MEB) and a dissemination barrier have been identified (Fig. 1) [32–34]. Since NS3/NS3a has a prominent role in virus release in insect cells *in vitro*, we now considered whether propagation of DISA vaccine virus, not expressing NS3/NS3a, would be significantly diminished or blocked in BTV-competent *Culicoides* biting midges *in vivo*. This blockade would improve the safety of replicating BT DISA vaccine by minimizing the risk on uncontrolled vaccine spread. Therefore, we here investigated the propagation of a DISA vaccine based on BTV1, expressing VP2 of serotype 2, after oral infection and intrathoracic injection of in *Culicoides sonorensis* (formerly *Culicoides variipennis sonorensis*) [35], a species known to vector several BTV serotypes including U.S. BTV2 [36].

## Methods

### Cells and viruses

BSR cells (a clone of BHK-21 cells [37]) were cultured in Dulbecco’s modified Eagle’s medium (DMEM, Invitrogen), with 5 % fetal bovine serum (FBS), 100 IU ml^−1^ Penicillin, 100 μg ml^−1^ Streptomycin and 2.5 μg ml^−1^ Amphotericin B, at 37 °C with 5 % CO_2_. KC cells [19] derived from embryos of colonized *Culicoides sonorensis* Wirth & Jones [38] were grown in modified Schneider’s Drosophila medium with 15 % FBS, 100 IU ml^−1^ Penicillin and 100 μg ml^−1^ Streptomycin at 27 °C.

BTV1 generated by reverse genetics (Genbank accession numbers FJ969719-FJ969727) with Seg-10 originating from BTV8 (AM498060), was used as virus backbone to generate BTV1 derivatives using reverse genetics as previously described [39, 40]. cDNA of Seg-2 of BTV2 (JN255863) was used for single Seg-2 exchange (BTV1[VP2]2). cDNA of Seg-10 originating from BTV8 with the out-of-frame deletion ΔC (bp 102–263) was used to generate NS3/NS3a knockout BTV with VP2 of serotype 2 (DISA 2) [41]. The positive control field strain of BTV11 was isolated from the spleen of a white-tailed deer from Texas in 2011, passaged once in embryonated chicken eggs, and four times in BHK-21 cells before use in midge feeding/injecting.

Virus stocks were produced by infection of BSR cells at low multiplicity of infection (MOI), and were harvested by freeze-thawing when > 50 % of cells immunostained as BTV-positive with α VP7 monoclonal antibody (MAb) ATCC-CRL-1875 in a duplicate well, or when > 50 % of cells showed cytopathogenic effect (CPE). Virus in clarified supernatant was concentrated using 3 K centrifugal filters (Amicon Ultracel-3 K) and centrifugation for 30 min at 4000 rpm. Seg-2 was confirmed by serotype specific PCR testing with primers targeting Seg-2 (BTV2 Seg-2 F 5′-TCAAAGATGAGGGGATACGG-3′ and BTV2 Seg-2 R 5′-AAGCGGCTGTTGATCCATAC-3′), and Seg-10 using PCR with Seg-10 primers (F-full-S10* 5′-GTTAAAAAGTGTCGCTGCC-3′ and R-full-S10 5′-GTAAGTGTGTAGTGTCGCGCAC-3′) followed by sequencing as described previously [31].

Virus titers were determined by endpoint dilution on BSR cells and expressed as 50 % tissue culture infectious dose per ml (TCID_50_ ml^−1^). The titer of the BTV1[VP2]2 virus stock was 10^6.4^ TCID_50_ ml^−1^, DISA 2 had a titer of 10^5.8^ TCID_50_ ml^−1^(intended dose for insect experiments was 10^6^ TCID_50_/ml). Wild type BTV11, used as a positive control, had a titer of 10^8.2^ TCID_50_ml^−1^.

CPE and protein expression of BTV1[VP2]2 and DISA 2 virus were determined using immunostaining of infected BSR monolayers with α VP7 MAb ATCC-CRL-1875 or with α NS3 MAb 31E9 (Ingenasa, Madrid, Spain) using standard procedures [42].

### *In vitro* virus release assay

Monolayers of KC cells (5 × 10^6^) or BSR cells (5 × 10^5^) in 2 cm^2^ wells were infected with an MOI of 0.01. Virus was adsorbed for 1.5 h at 27 °C or 37 °C for KC and BSR cells respectively. Unattached virus was removed by washing with PBS, and fresh medium was added. This time point was set as 0 h post-infection (hpi). Incubation was continued and cells and culture media were harvested at indicated time points. Cells were lysed to be able to study the intracellular BTV fraction, by freeze thawing at −80 °C. Virus titers were determined and growth experiments were independently repeated.

### Insect feeding and sampling

Colonized 3–4 day old female *C. sonorensis* midges from the Arthropod-Borne Animal Diseases Research Unit, Manhattan, KS, USA [43] were offered a blood meal, consisting of 1:1 (v/v) defibrinated sheep blood and virus stock at highest titer available, in an artificial feeder using a parafilm membrane [44]. Midges were allowed to feed for 2 h, removed from the blood source, anesthetized for 10–15 s with CO_2_, removed from the feeding cage, and sorted as to blood-feeding status on a CO_2_ fly pad (Diamed Lab Supplies, Inc., Mississauga, Ontario, CA). Engorged females were put in cardboard cages with cotton-plugged vials containing 10 % sucrose and held at 26 °C. At 0, 7, 10 (BTV11 control only) and 14 days post feeding (dpf), 50 midges were anesthetized with CO_2_and heads were separated from bodies using ultra-fine tweezers (EMS Hatfield, PA, USA) and a dissecting microscope (SMZ 1500; Nikon Instruments, Melville, NY, USA). Heads and bodies were separately placed in 100 μl RNAlater (Qiagen, Germantown, MD, USA), and stored at 4 °C.

### Insect inoculation

Colonized 3–4 day old female *C. sonorensis* midges were injected intrathoracically with 46 nl of virus stock using the Nanoject II microinjector (Drummond Scientific, Broomall, PA, USA) under a dissecting microscope (Nikon Instruments). This volume was based on hemocoel injection capacity of the midges, highest rate of consistency, complete fluid retention, and maximum post inoculation survival. Total virus injected per midge, based on highest starting virus stock titers possible, was 10^1.5^ TCID_50_ of DISA 2 and 10^2.1^ TCID_50_ of BTV1[VP2]2 virus. Inoculated midges were placed in cardboard cages with cotton-plugged vials containing 10 % sucrose and held at 26 °C. At 0, 7, and 10 days post inoculation, 25 midges were anesthetized, decapitated and stored in RNA later (Qiagen) at 4 °C as described above. Time 0 samples were taken from 1 to 4 h post-injection. This variation was due to the time-intensive nature of microinjecting large numbers of midges needed to ensure adequate numbers of surviving midges for each time point.

### RNA isolation and PCR

PBS (400 μl) and one 5 mm stainless steel ball (Qiagen) were added to midge bodies and heads in RNA later in micronic tubes. Tubes were shaken for 3 min at 50 Hz in a tissue lyser (85600, Qiagen). After centrifugation, 200 μl of supernatant was used for RNA isolation using the MagNApure 96 DNA and viral NA Small Volume kit (Roche) by the MagNApure isolation robot (Roche) according to the manufacturer’s protocol [45]. The real-time RT-PCR test for Seg-1 was performed using primer F-pan-S1 (5′-TTAAAATGCAATGGTCGCAATC-3′), primer R-pan-S1 (5′-TCCGGATCAAGTTCACTCC-3′) and probe P-pan-S1 (5′-6-FAM-CCGTGCAAGGTGC-MGB-3’) [46] according to the all-in-one method, including the pre-denaturation step, as described for the pan BTV Seg-10 PCR test [45]. Crossing point (*Cp*) values of each PCR were calculated, and negative results were arbitrarily set as 45 to allow these to be included in the analysis. Due to the maximum of 45 cycles, the highest *Cp* value that could still be calculated was 40. Statistical differences in *Cp* values were calculated using a one way ANOVA and subsequent Tukey’s multiple comparison test, with *p* < 0.001 indicating significant differences between days or groups.

## Results

### NS3/NS3a is required for release from *Culicoides* cells *in vitro*

**Fig. 2:**
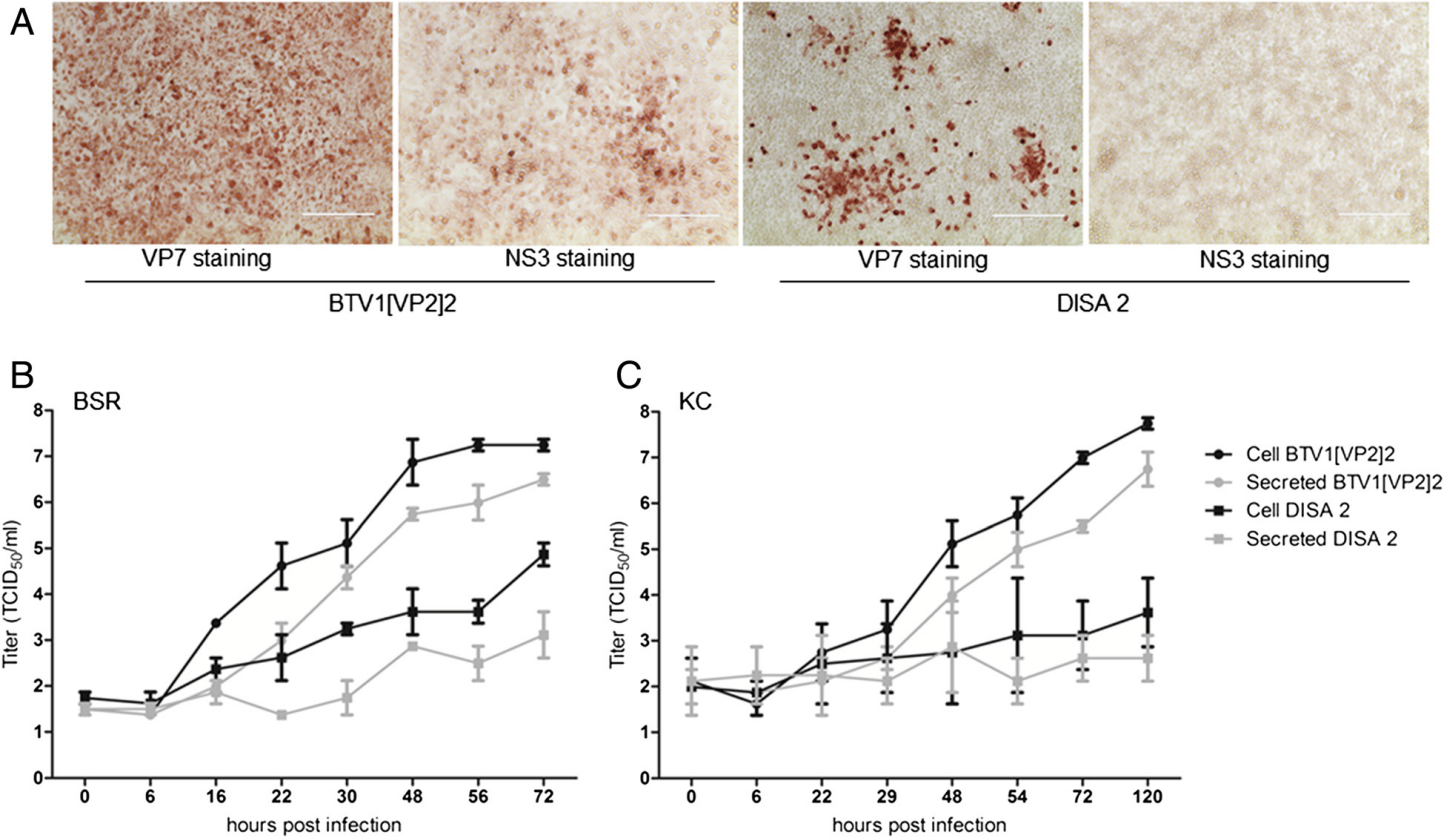
BSR monolayers were infected with BTV1[VP2]2 or DISA 2 and immunostained with α NS3 or VP7 MAbs. Clearly, VP7 is expressed in both infections (red colour), whereas NS3/NS3a is only detectable in the BTV1[VP2]2 infected cells (**a**). Release of BTV1[VP2]2 and DISA 2 virus on BSR cells (**b**) and KC cells (**c**) was also examined. Both virus in the cell fraction (black) and released virus (grey) was measured. Experiments were performed in duplicate and error bars represent SEM

Similar to previous results with NS3/NS3a knockout mutant viruses [28], DISA 2 did not induce CPE in BSR cells. Immunostaining of VP7 indicated BTV replication, but NS3/NS3a expression could not be detected. In contrast, BTV1[VP2]2 parental virus induced CPE and was immunostained with both VP7 and NS3 MAbs (Fig. 2a). Sequencing of Seg-10 of DISA 2 confirmed the ΔC deletion, and thus the absence of NS3/NS3a expression by DISA 2. No additional insertions or deletions in Seg-10 of DISA 2 were identified (not shown). In agreement to previous results [28], release of DISA 2 from BSR cells was slightly delayed compared to the parent virus, with clear increase examined at 48 h post infection, compared to 16 h post infection for BTV1[VP2]2 (Fig. 2b). The virus titer in the cells was higher for BTV1[VP2]2 (10^7.2^ TCID_50_ ml^−1^) compared to DISA 2 (10^4.9^TCID_50_ ml^−1^) at the end of the experiment at 72 h post infection. Release of virus was also reduced for DISA 2 (10^3.1^ TCID_50_ ml^−1^) compared to the parental virus (10^6.5^TCID_50_ ml^−1^). Growth in KC cells was highly attenuated, with ten-fold lower virus titer in both the cell fraction and the released fraction for DISA 2 at the end of the experiment. DISA 2 virus release was diminished, with 10^2.1^ TCID_50_ ml^−1^ at 0 dpi and still only 10^2.6^ TCID_50_ ml^−1^ at 120 dpi, the end of the experiment (Fig. 2c).

### *C. sonorensis* midges can be infected orally with BTV11, but not with BTV1[VP2]2

**Fig. 3:**
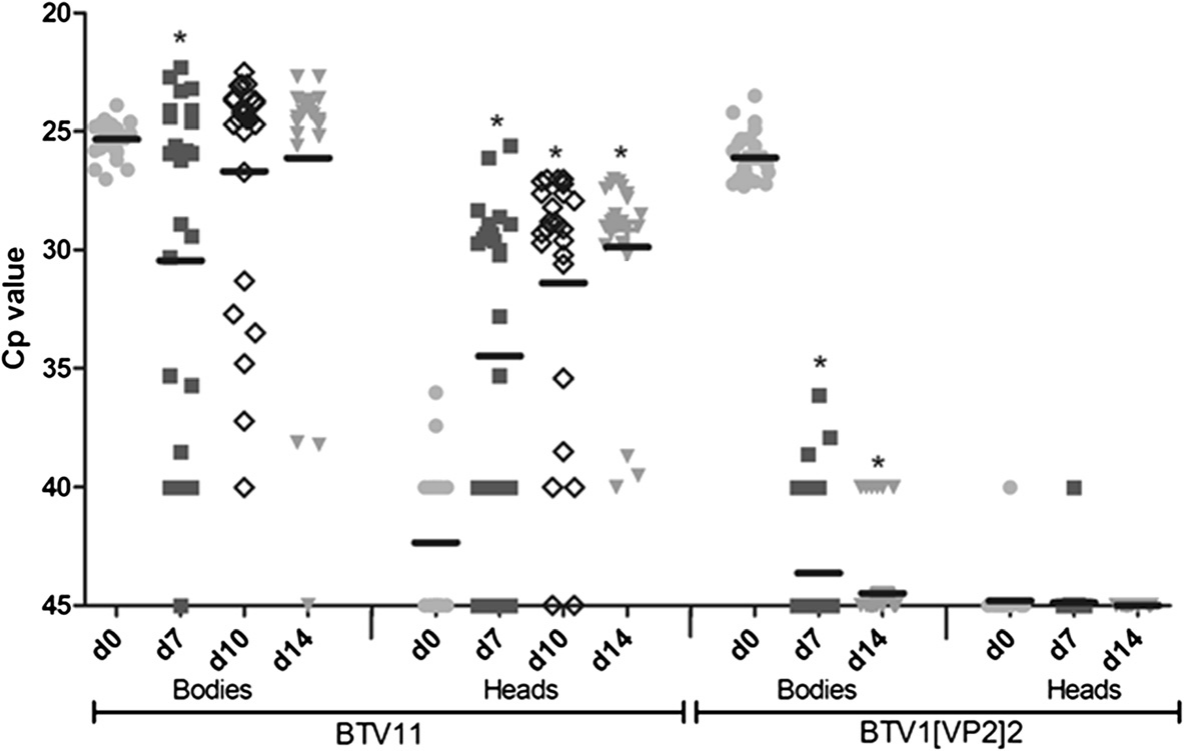
Colonized *C. sonorensis* were fed with blood containing BTV11 or BTV1[VP2]2. Viral RNA was detected and semi quantitated by PCR expressed in *Cp* values for individual heads and bodies at day 0 (light grey dots), day 7 (dark grey squares), day 10 (open diamonds) and day 14 (light grey triangles) post feeding. The mean *Cp* values (black line) and significant (*p* < 0.001) different *Cp* values from the respective day 0 value (*) are indicated

BTV11-fed midges were harvested at day 0, 7, 10 and 14 dpf. For each day of sampling, heads and bodies of 25 midges were harvested and were tested individually. Viral RNA was isolated for PCR testing and semi-quantitated by *Cp* values (Fig. 3). Directly after feeding (day 0), all bodies were PCR positive (mean *Cp*: 25.3, *range 27–23.9*), whereas heads were negative (13 out of 25) or had very high *Cp* values (mean *Cp*:39.5). As blood meals are digested after 3 dpf, the *Cp* value at 7dpf in the bodies (mean *Cp*: 30.5, *range 45–22.3*), was slightly higher, but decreased significantly until day 10 (mean *Cp*: 26.7, *range 40–22.5*) (*p* < 0.001), indicating virus replication in the midge. From day 10 to 14 (*Cp*: 26.1, range 45–22.7), no significant difference was found, suggesting no further increase of the virus titer in midge bodies. To study the ability of BTV11 to cross the midgut barrier and to replicate in the midge, viral RNA in the heads was determined. *Cp* values were significantly lower at day 7 (mean *Cp*: 34.5,*range 45–26.1*), day 10(mean *Cp*: 31.4,*range 45–27*);and day 14 (mean *Cp*:29.9,*range 40–26.7*) compared to day 0 (*p* < 0.001) and were declining in time. This clearly demonstrated midgut escape and virus replication in colonized *C. sonorensis* by BTV11.

Similarly, 50 BTV1[VP2]2 fed midges were harvested at day 0, 7 and 14, and tested by PCR. At day 0, directly after feeding, bodies showed *Cp* values (mean *Cp*: 26.1, *range 27–23.5*) not significantly different from that of bodies of BTV11 fed midges. At day 7, 40 out of 50 bodies were PCR negative, and at day 14 post feeding only 6 out of 50 bodies had a *Cp* value of 40. All heads were PCR negative at day 0, 7 and 10, except for one head at day 0 and 7 (*Cp*:40). These results indicated that BTV1[VP2]2 was unable to infect the midgut epithelium and was unable to escape the midgut barrier in *C. sonorensis*. Feeding with DISA 2 virus was also performed, but harvested heads and bodies were not further processed because of the negative results of the BTV1[VP2]2 parental strain.

### NS3/NS3a is required for propagation of artificially disseminated BTV in *C. sonorensis*

**Fig. 4:**
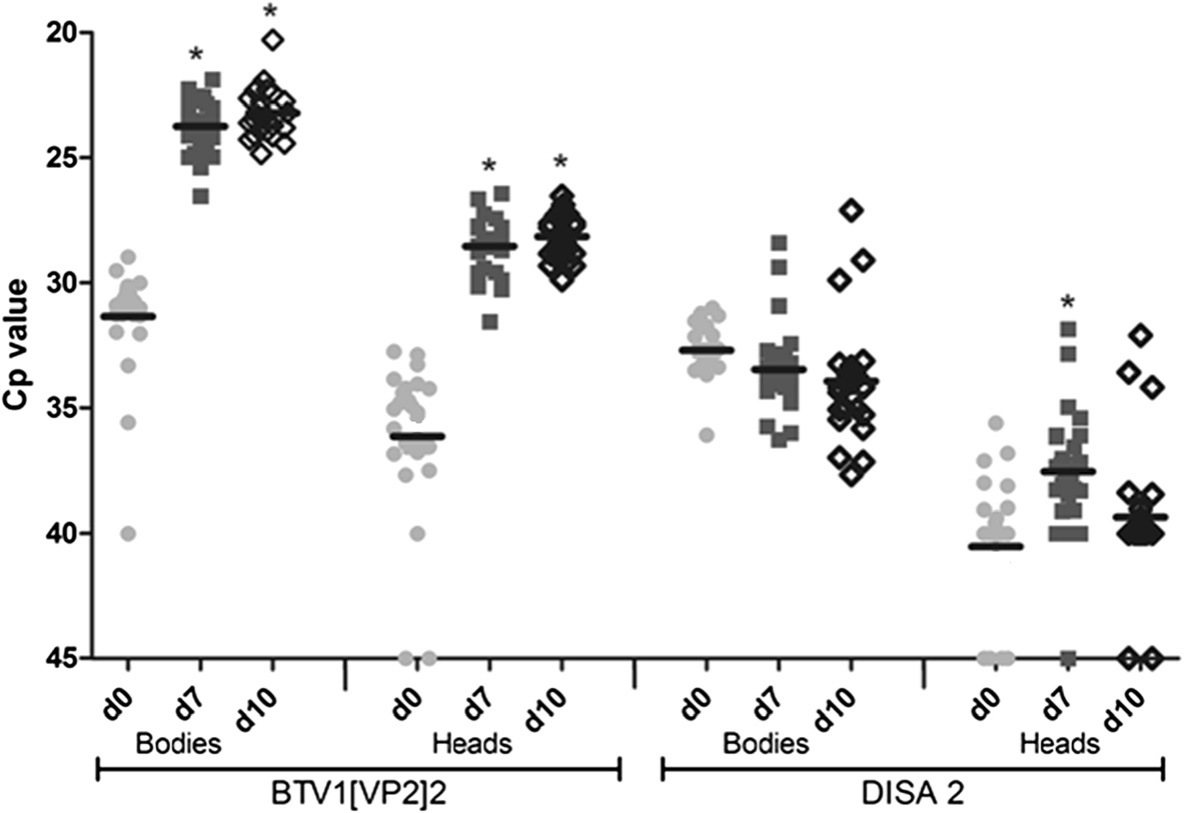
Colonized *C. sonorensis* were injected with BTV1[VP2]2 or DISA 2.Viral RNA was detected and semi quantitated by PCR expressed in *Cp* values for individual heads and bodies indicated at day 0 (light grey dots), day 7 (dark grey squares) and day 10 (open diamonds) post injection. The mean *Cp* values (black line) and significant (*p* < 0.001) different *Cp* values from the respective day 0 value are indicated (*)

Intrathoracic injection of virus into the hemocoel of midges can lead to replication of virus unable to naturally escape the midgut following oral uptake [32, 47]. Therefore, to study virus propagation in the absence of a midgut barrier, midges were injected with the parent strain BTV1[VP2]2 and its NS3/NS3a knockout derivative DISA 2. Groups of 25 injected midges were harvested at day 0, 7, and 10 post injection. Viral RNA in heads and bodies was individually tested and semi-quantitated by PCR (Fig. 4).

On day 0, RNA was detected in all bodies for BTV1[VP2]2 (mean *Cp*: 31.3, range 40–30) and DISA 2 (mean *Cp*: 32.7, range 36.1-31). There was no significant difference between both groups. Injected virus was also detected in the heads of 23 out of 25 midges on day 0 due to dissemination throughout the hemocoel. At both day 7 and 10 post injection of BTV1[VP2]2, mean *Cp* values in the bodies were 23.7 and 23.2 (*range: 26.5-21.9 and 24.4-20.3* respectively), indicating significantly higher RNA levels than that of input virus. In addition, *Cp* values of tested heads on these days were also significantly lower (p < 0.001) (28.5 and 28.2 with a *range of 31.5-26.4 and 29.9-26.5*, respectively) compared to day 0 (mean *Cp*: 36.1). These results clearly indicate virus propagation of BTV1[VP2]2 throughout injected *C. sonorensis* midges.

In contrast, DISA 2 injected midges did not show significantly lower *Cp* values in the bodies in time, with mean *Cp* values of 32.7, 33.5 and 33.9 for day 0, 7, and 10, respectively. In the heads of DISA 2 injected midges, *Cp* values were significantly lower (*p* < 0.001) at day 7 (mean *Cp*: 37.5, range 45–31.8) compared to day 0 (mean *Cp*: 40.5 range 45–35.6). However, at day 10, the mean *Cp* value was not significantly different from day 0 (mean *Cp*: 39.3, range 45–32.1). Remarkably, even at the day 0 time point (1–4 hpi), DISA 2 injected midges had a higher mean *Cp* value of 40.5 (range: 45–35.6) in their heads compared to BTV1[VP2]2 (mean *Cp:* 36.1, range: 45–32.7). Unlike the parent BTV1[VP2]2 virus strain, DISA 2 was unable to propagate after injection of colonized *C. sonorensis*. Because these viruses only differ in NS3/NS3a expression, we conclude that NS3/NS3a proteins play a crucial role in virus propagation in the insect vector.

## Discussion

Recently, we have shown that NS3/NS3a is not essential for virus replication *in vitro* [28]. A novel BT vaccine candidate based on live-attenuated BTV lacking NS3/NS3a expression has been developed and is named the disabled infectious, single animal (DISA) vaccine [28–31, 41]. NS3/NS3a expression is more prominent in insect cells [19–23], and important for virus release from cultured midge cells *in vitro* [28]. Here, the role of NS3/NS3a in virus propagation was studied *in vivo* in midges.

We compared a reverse genetics derived parent strain BTV1[VP2]2 (BTV1 expressing VP2 of serotype 2) with the same virus containing a deletion in Seg-10, which abrogates NS3/NS3a expression (DISA 2). Virus growth of DISA 2 in mammalian cells (BSR) is inhibited by the NS3/NS3a knockout mutation, and is delayed (Fig. 2b). Both release and growth of DISA 2 in *Culicoides* cells is strongly attenuated by the NS3/NS3a knockout mutation (Fig. 2c). BTV1[VP2]2 and DISA 2 grow to lower titers in BSR cells, compared to similar viruses with other serotypes, as already observed in earlier studies [30]. So apparently, serotyping with VP2 from serotype 2 influences *in vitro* growth in a negative manner, likely by disturbing infection.

Initially, we compared oral infection of *C. sonorensis* with BTV1[VP2]2 and an American BTV11 isolate. This BTV11 isolate was included to confirm ‘normal’ feeding behaviour of this specific midge colony and was fed with a very high dose of 10^8.2^ TCID_50_ ml^−1^, since this dose led to virus replication in numerous previous experiments (not shown). Unfortunately, the BTV1[VP2]2 strain was not able to escape the midgut barrier as evidenced by the lack of viral RNA in the heads. The results of day 0 post feeding suggests a similar starting dose in the midguts of both groups by PCR testing. However, the blood meal for BTV1[VP2]2 contained 100 times less infectious virus than that of BTV11 as determined by titration. This might be explained by the presence of non-infectious particles in the BTV1[VP2]2 virus stock, although this needs further investigation. The lower dose could explain the negative results for BTV1[VP2]2, but a similar dose of about 10^6^ TCID_50_ ml^−1^ of BTV1, as used in our study for BTV1[VP2]2,has been used already successfully in several vector competence studies using *C. sonorensis* [32, 33, 48, 49]. Therefore, it is highly unlikely that the used dose is too low to be able to infect the midges and it is not expected that a higher dose would have led to BTV1[VP2]2 replication. In further support of this conclusion, intrathoracic injection of only 46 nl of the same virus stock resulted to similar *Cp* values at day 0, proficient for virus replication, while it is estimated that fully engorged *C. sonorensis* midges ingest approximately 50–100 nl of blood (personal communication B. Drolet, U.S. Department of Agriculture). This volume correlates to 50–100 TCID_50_ of virus per blood meal when feeding with viremic blood containing 10^6^ TCID_50_ of BTV ml^−1^.

Only a very few *Culicoides* species have been shown as transmission-competent vectors for various serotypes of BTV [32, 50–53]. Vector competence for a specific arbovirus is affected by multiple factors during the virus-vector interaction, including viral genetics, vector genetics, gut microbiota, physiological barriers, salivary components, environmental temperature, and vector innate immunity [32, 33, 54–57]. Physiological barriers include the MIB, MEB, salivary gland infection barrier and salivary gland escape barrier. For bite transmission of virus after ingestion of BTV1[VP2]2 or DISA2 strain, viruses must exit the midgut (with or without infection), enter the hemocoel, disseminate, and then infect and escape the salivary glands. Based on the PCR results of bodies and heads of orally infected midges, BTV1[VP2]2could not overcome the MIB or MEB, since no virus replication in the bodies and no dissemination to heads was observed. This strongly indicates that, *C. sonorensis* is not competent for this recombinant virus. No propagation of parental NS3/NS3a expressing strains in the midge vector could make the DISA vaccine even safer regarding vaccine virus spread in the field. Intrathoracic inoculation was performed to circumvent the midgut barriers and to study the ability of disseminated BTV1[VP2]2 and DISA 2 to propagate within *Culicoides*.

Replication of BTV is likely after intrathoracic injection, even for viruses that do not replicate in the midge after oral uptake [32]. Indeed, in contrast to oral uptake, BTV1[VP2]2 propagated in injected midges. As expected, DISA 2 virus was detected in the heads directly after injection due to dissemination in the hemocoel. The amount of DISA 2 virus RNA was significantly less in heads (*p* < 0.001) (higher *Cp*), compared to BTV1[VP2]2 at day 0. This might be due to possible variations in exact sampling times post inoculation for the day 0 time point (1–4 hpi) or differences in amount of virus delivered in the 46 nl injection (10^1.5^ vs. 10^2.1^). Less time between injection and decapitation could lead to incomplete dissemination of virus to the head via the hemolymph. Significantly higher DISA 2 RNA levels (lower *Cp* value) were measured in the heads at day 7 compared to day 0, which is likely due to full dissemination of injected virus at this time point. Indeed, the *Cp* value at day 10 was higher again compared to day 7, suggesting degradation of viral RNA. A few individual bodies and heads of DISA 2 fed midges showed a *Cp* value lower than the average at day 7 and 14. This might indicate a low level of replication in these midges. However, compared to BTV1[VP2]2 fed midges, *Cp* values are still high, and spread via the salivary glands to the ruminant host is highly unlikely.

## Conclusions

In conclusion, NS3/NS3a knockout strongly reduces virus release and propagation in the midge after injection, once the midgut barrier has been passed. This is likely due to the strongly reduced virus release, also seen *Culicoides* cells. It is also possible that the delayed release of DISA 2 from the cell results in faster virus clearance by intracellular innate immunity such as RNAi [58, 59], Toll-Imd, Jak-STAT, NfkB, and autophagy [60].

The virus backbone used in DISA vaccines is based on non-virulent BTV and is safe with respect to fever and clinical signs [61]. Currently, the DISA principle is based on the absence of viremia in the vertebrate host, thereby preventing uptake by insect vectors [29]. Here, the safety of the DISA principle is supported by the absence of DISA 2 propagation in *C. sonorensis.*

Live-modified vaccines for orbiviruses potentially imply several risks, such as reversion to virulence, re-assortment events and uncontrolled spread of vaccine virus. An elegant method to combat these major disadvantages is to target functions that are specific and essential for virus propagation in the insect vector, and for virus transmission between host and vector. Most likely, similar NS3/NS3a deletion mutants for other orbiviruses will have similar characteristics [62], whereas targeting functions essential for vector transmission in other arboviral pathogens will also result in safe vaccine candidates.
